# How does sugar-sweetened beverage consumption relate to sleep and mental health in adolescents? A scoping review

**DOI:** 10.3389/fnut.2025.1718230

**Published:** 2026-01-12

**Authors:** Annalisa Di Nucci, Erica Cardamone, Laura Rossi, Marco Silano

**Affiliations:** 1 Department of Cardiovascular, Endocrine-Metabolic Diseases and Aging, Italian National Institute of Health, Rome, Italy; 2 Department of Medicine, University of Udine, Udine, Italy; 3 Unit of Human Nutrition and Health, Department of Food Safety, Nutrition and Veterinary Public Health, Italian National Institute of Health, Rome, Italy

**Keywords:** adolescents, diet, food intake, mental health, sleep, sugar-sweetened beverages

## Abstract

**Introduction:**

Over the last years, adolescents have exhibited high consumption of sugar-sweetened beverages (SSBs). During this developmental stage, characterized by profound biological and psychosocial changes, individuals are more susceptible to sleep disturbances, as well as psychological, behavioral, and emotional difficulties. The present scoping review aims to offer insights into how SSBs consumption relates to sleep and mental health outcomes in adolescents.

**Methods:**

A systematic search and selection process was conducted across four electronic databases. Studies, published in peer-reviewed international scientific journals up to April 2025, in English, and examining the relation between SSBs, sleep, and mental health in adolescents were considered eligible.

**Results:**

The search identified 288 references after duplicate removal. Based on PCC framework, 57 studies were included. Slightly fewer studies investigated the association between SSBs consumption and sleep outcomes (*n* = 25), compared with those focusing on mental health outcomes (*n* = 32). Evidence suggests a potential link between SSBs consumption, sleep, and mental health, indicating that higher intake may be associated with increased sleep disturbances and mental health problems.

**Discussion:**

Overall, the results of this review advance the hypothesis of a possible bidirectional relationship between SSBs consumption and both adverse sleep and mental health outcomes. These findings should be interpreted with caution, due to the main gaps identified. The current evidence calls for future studies that use interventional and longitudinal designs, focus on adolescence, target regions with rising SSBs consumption and sleep or mental health issues, analyze SSBs subgroups separately, and address all sleep dimensions. This review also highlights the need for tailored public health intervention strategies that address all lifestyle domains relevant to adolescent health.

**Scoping review registration:**

https://osf.io/kzu7y/overviewn, DOI: 10.17605/OSF.IO/KZU7Y.

## 1 Introduction

Sugar-sweetened beverages (SSBs) are defined as a broad category of drinks containing free sugars, such as soft drinks, certain fruit juices, energy drinks (EDs), and some caffeinated beverages (1).

Children and adolescents exhibit the highest consumption of SSBs across population groups (2), with these beverages representing the predominant source of free sugars in their diet (3, 4).

The widespread use of SSBs in childhood has been recognized as a major public health concern worldwide (5) and has been targeted through national and international policies and strategies aimed at counteracting its spread (1). Consistently, several nutritional guidelines advise a limited consumption of SSBs in pediatric age (6, 7), in line with the World Health Organization (WHO) recommendation to reduce free sugars to less than 10% of total energy intake across the life course to prevent obesity, its complications, and dental caries (8).

Nevertheless, a recent population-based study including 185 countries reported a 23% increase (0.68 servings per week) in SSBs consumption among children and adolescents from 1990 to 2018. Higher intakes were observed in older versus younger participants, those resident in urban versus rural areas, and those with parents of higher versus lower educational attainment, highlighting the need to address the determinants of SSBs consumption especially in adolescents and socially vulnerable groups (2).

A variety of factors encompassing economic, individual, behavioral, and environmental drivers could explain this phenomenon (9). Evidence suggests that the accessibility and availability of SSBs, together with exposure to food advertising through media use, are associated with higher consumption. Parental modeling and peer influence also play key roles in determining adolescents’ beverage choices (9, 10).

High consumption of SSBs significantly contributes to overall energy intake, promoting weight gain, and playing a central role in the childhood obesity epidemic and associated metabolic diseases (3, 11, 12). Consequently, most research on SSBs so far has focused on their impact on these conditions (3, 4, 13, 14). However, in recent years, a growing body of evidence indicates the relevance of additional pediatric health challenges, particularly in adolescence, including sleep problems and mental health issues (15, 16).

Epidemiological studies have revealed that sleep problems are highly prevalent among adolescents, affecting around 40% in Europe and Asia and about one-third of children and adolescents in the United States (17, 18). Although lower than the sleep problems, the Global Burden of Disease Study estimates a still concerning prevalence of about 13–14% for mental health disorders in adolescents, with depression and anxiety being the most common (16). These two conditions are closely interconnected and often influence each other. Systematic reviews and meta-analyses have shown that insufficient or poor-quality sleep is associated with an increased risk of depression, anxiety, and other emotional difficulties in adolescents (19, 20). Conversely, mental health problems, particularly internalizing disorders, can exacerbate sleep disturbances, suggesting a bidirectional relationship between these domains (21, 22).

Dietary habits can influence both sleep and mental health outcomes in adolescents, with unhealthy patterns being associated with poorer sleep quality and increased emotional difficulties (3, 23). Among dietary factors, SSBs appear particularly relevant, as higher consumption has been linked to shorter sleep duration, lower sleep quality, and elevated risk of depressive and anxiety symptoms, thereby contributing significantly to the complex interplay between sleep and mental health outcomes (3, 24, 25).

Overall, the high prevalence of SSBs consumption in adolescents, together with their increased vulnerability to sleep and emotional regulation problems, underscores the need to examine the relationship between these factors specifically in this age group. Indeed, adolescence is characterized by profound neurobiological and psychosocial changes, including greater autonomy over dietary choices and a pubertal shift toward later sleep timing, which may amplify the potential effects of SSBs on both sleep and emotional regulation (26, 27). These issues need to be addressed separately from younger children to develop effective targeted prevention strategies.

Despite existing research, no study has yet provided a comprehensive overview of the mechanisms linking SSBs intake with sleep and mental health outcomes in this age group. This study therefore aims to fill this gap and offer insights into how SSBs consumption relate to these outcomes, by addressing the following research questions: (1) Is there an association between SSBs consumption and sleep and mental health outcomes in adolescents? (2) Does SSBs consumption affect sleep and mental health? Is the opposite also true? (3) What are the potential mechanisms underlying this relationship?

## 2 Materials and methods

A Scoping Review was conducted using the Preferred Reporting Items for Systematic Reviews and Meta-Analyses extension for scoping review (PRISMA-ScR) guidelines (28) to guide the search and identification process.

The protocol was registered in the Open Science Framework (OSF) database (registration DOI: 10.17605/OSF.IO/KZU7Y).

### 2.1 Eligibility criteria

The PCC (Population, Concept, Context) framework followed to establish eligibility criteria is presented in Table 1. According to the WHO definition of adolescence (29), the age range of 10–19 years was selected as an inclusion criterion. Studies that analyzed the consumption of SSBs and its relationship with mental health and sleep-related outcomes were included. All types of SSBs (e.g., caffeinated drinks, energy drinks), and mental health and sleep-related outcomes were considered eligible.

**Table 1:** Population, concept, context (PCC) framework.

Parameter	Description
Population	*Inclusion:* Adolescents (10–19 years. old)
	*Exclusion:* Children (<10 years old), adults (>19 years old), elderly (>70 years old)
Concept	*Inclusion*: SSBs consumption; Mental health outcomes; Sleep-related outcomes
	*Exclusion*: Dietary patterns that included SSBs consumption; Other health-related outcomes
Context	*Inclusion*: Every setting and geographical context
	*Exclusion:* None
Types of evidence sources	*Inclusion*: Observational studies (prospective cohort studies, cross-sectional studies and case–control studies) and RCTs, published in peer-reviewed international scientific journals up to April 2025, in English
	*Exclusion*: Studies not published in peer-reviewed international scientific journals, not in English

SSBs, Sugar-Sweetened Beverages; RCTs, Randomized Clinical Trials.

No restrictions were applied to the setting and geographical context.

The criteria for excluding an article were: not targeted at adolescents, focused on the analysis of dietary patterns that included SSBs consumption (without analyzing individual food groups) and on health outcomes other than mental health and sleep, not published in peer-reviewed international scientific journals, not available in English.

### 2.2 Search strategy

A literature search was performed using four electronic databases: MEDLINE (PubMed), Embase, Scopus, and Web of Science. Using relevant subject headings and free text search terms, the search strategy was based on the following keywords: ‘sugar-sweetened beverages’, ‘sugary beverages’, ‘sugary drinks’, ‘adolescent’, ‘teenager’, ‘youth’, which were used in combination with words relating to mental health and sleep outcomes, including ‘sleep hygiene’, ‘sleep quality’, ‘sleep duration’, ‘sleep disorders’, ‘mental health’, ‘psychological symptoms’, and their variants. The search strategies are available in Supplementary Table S1.

### 2.3 Study selection

Two reviewers (A.D.N., E.C.) selected and screened the studies to be included in this review. Duplicate studies were removed using the software system Rayyan (30). No formal pilot testing was conducted. However, the screening criteria were discussed among the reviewers at a preliminary phase, in order to adjust the procedure. All identified articles were initially screened by title and abstract. The reviewers were blinded to each other’s decisions and disagreements between individual judgements were resolved by consensus. Applying the eligibility criteria, the articles were subsequently screened by full text, jointly by the reviewers. The software systems used for managing articles were Microsoft Excel 2016 and Mendeley (Mendeley Reference Manager v2.137.0, Elsevier, London, UK).

### 2.4 Data extraction

Data extraction was performed by two reviewers (A.D.N., E.C.) in parallel. Uncertainties were resolved by the reviewers through discussion.

The following data were extracted from each eligible paper, using a data extraction tool developed by reviewers: first author’s last name; year of publication; study design; country; number and age of participants; objective; and main results (adjusted for confounders, where possible).

Studies that included both children and adolescents, as well as those that classified individuals aged 18–19 years as adults, were considered to ensure that no adolescent-related findings were excluded. For the same reasons, papers that did not analyze data by age subgroups were also included. However, whenever possible, information on the size of the adolescent subsamples was extracted. Finally, studies that assessed SSBs consumption alongside other relevant dietary variables (e.g., fast-food consumption) and studies that examined the mediating role of SSBs in the relation between health outcomes of interest were included.

## 3 Results

**Figure 1:**
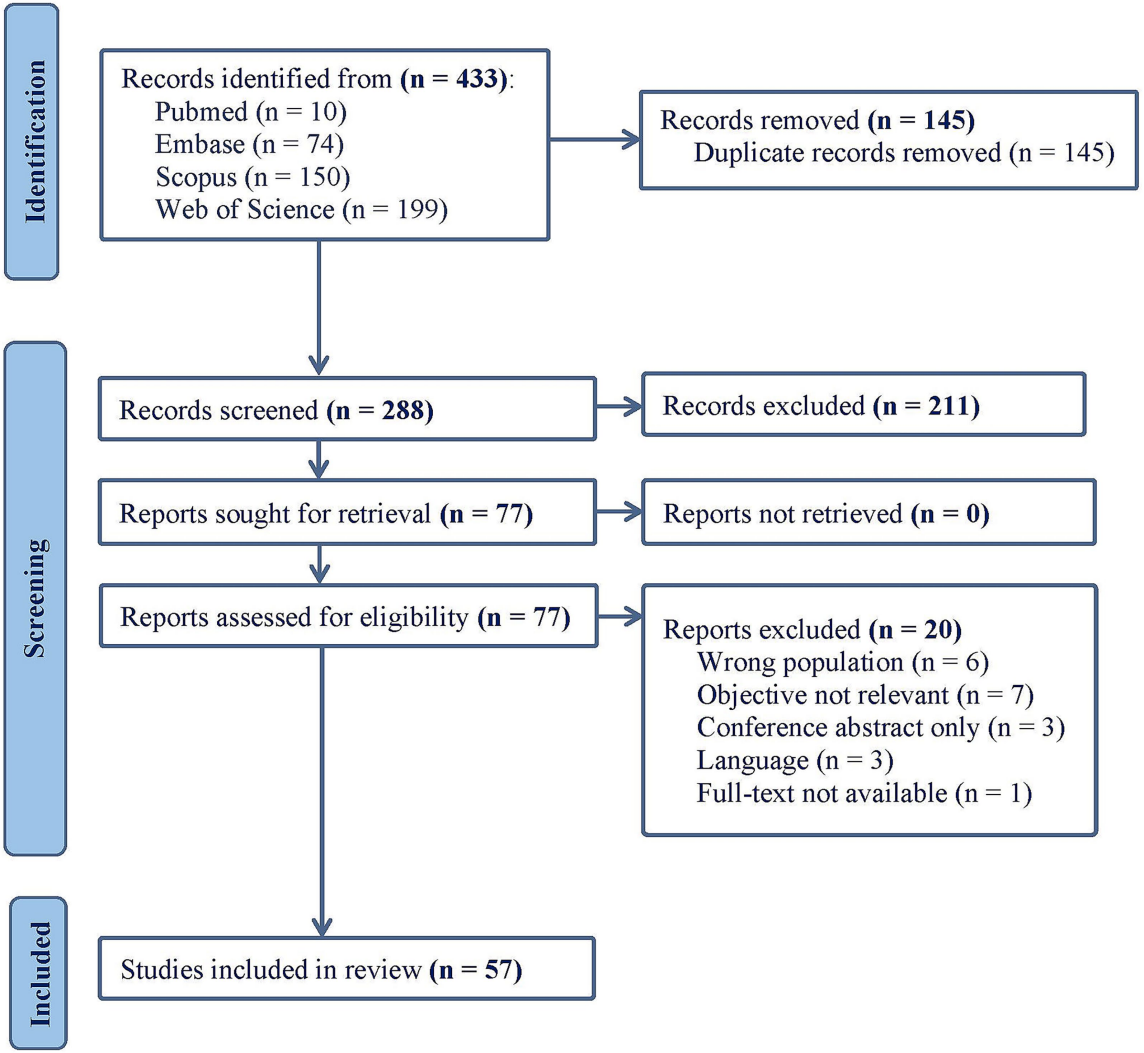
PRISMA flow chart.

The process of study selection is summarized in Figure 1. A total of 433 articles were initially retrieved from four electronic databases. After removing 145 duplicate records, 288 articles remained for title and abstract screening, which led to 77 studies being selected for full-text screening. Of these, 20 articles were excluded for not meeting the inclusion criteria (see Supplementary Table S2), resulting in a final set of 57 studies included in this scoping review. Data extraction with key characteristics of these studies are presented in Tables 2, 3. Most of the included studies had a cross-sectional design (*n* = 48), followed by cross-sectional and longitudinal (*n* = 4), longitudinal (*n* = 3), and the remaining two with an interventional study design.

**Table 2:** Characteristics of the studies addressing sleep that were included in the review.

Continent	Articles	Study design	Country	Population Sample size (N) and age (mean ± standard deviation)	Objective	Main results
Asia	Almulla A. et al. (2020) (32)	Multi-center cross-sectional	United Arab Emirates	1,611 10–18 y (13.5 ± 2.3)	To investigate the prevalence of EDs consumption and its relationship with socio-demographic characteristics, eating habits, BMI, SD, PA, and ST.	EDs consumers were less likely to meet sleep duration recommendation (39% vs. 61%, *p* < 0.001). Students not meeting sleep recommendation duration were 1.5 times more likely to consume EDs (AOR = 1.47; 95% CI: 1.14, 1.89; *p* = 0.003).
	Boozari B. et al. (2021) (33)	Cross-sectional	Iran	395 18–43 y (22.8 ± 3.9) ≤23 y: 261 (66.08%) >23 y: 134 (33.92%)	To evaluate the relationship between SD and SQ with sugar and SSBs intake.	Short sleepers (<6 h/day) had higher consumption of SSBs intake (86.54 vs. 65.73 g/day; *p* = 0.05) in comparison with those who had more than 8 h/day of sleep. Poor quality sleepers had significantly higher intake of SSBs compared with those with good quality of sleeping (87.09 vs. 56.73 g/day; *p* = 0.004). No significant correlation was found between SD and SSBs intake. SQ score was positively correlated with SSBs intake (rp:0.14, *p* = 0.007) in whole population. Similar results were found in younger individuals (rp:0.27, *p* = 0.002) and non-obese participants (rp:0.14, *p* = 0.006).
	Gan W. et al. (2019) (54)	Cross-sectional	Malaysia	421 12–16 y (13.3 ± 1.3)	To assess the associations of lifestyle and socio-environmental factors, and body weight status with consumption of SSBs.	Simple linear regression showed that poorer SQ (*β* = 0.196; *p* < 0.001) was associated with higher SSBs intake. This association remained significant in multiple linear regression (*β* = 0.228; *p* < 0.001).
	Huang Z. et al. (2025) (34)	Cross-sectional	China	186,723 6–18 y 6–12 y (1st-6th grade): 86,254 (46.19%) 12–15 y (7th-9th grade): 57,938 (31.03%) 15–18 y (10th-12th grade): 42,531 (22.78%)	To investigate sleep status and its association with dietary habits.	The univariable analysis revealed that times of SSBs consumption per week were significantly associated with the sleep status (X^2^ = 16.322; *p* < 0.001). Results of multivariable logistic regression analysis showed that who consumed SSBs 4 ~ 5 times/week (OR = 2.066; 95% CI: 1.903–2.243) or >5 times/week (OR = 2.021; 95% CI: 1.838–2.223) had significantly higher odds of insufficient sleep compared to those with SSBs consumption <4 times/week (*p* < 0.001).
	Li W. et al. (2018) (35)	Cross-sectional	China	788 19.8 ± 1.1 y 18–19 y (grade 1): 212 (26.9%) 19–20 y (grade 2): 255 (32.4%) 20–21 y (grade 3): 321 (40.7%)	To explore the key mediating role of SSBs consumption in the association among SD, late chronotype, and weight gain.	The significant indirect effect of SSBs consumption was found between chronotype and BMI (effect = − 0.03, SE = 0.01, 95% CI [− 0.05, − 0.02]) and between SD and BMI (effect = − 0.12, SE = 0.05, 95% CI [− 0.16, − 0.09]). In addition, physical exercise and psychological condition also play mediating effects between chronotype and BMI (effect = − 0.04, SE = 0.01, 95% CI [− 0.06, − 0.01] and effect = − 0.03, SE = 0.02, 95% CI [− 0.05, − 0.01]), but their mediating effect was not found between SD and BMI.
	Liu A. et al. (2022) (36)	Cross-sectional	China	5,254 6–17 y 6–12 y: 3,573 (68.01%) 13–17 y: 1,681 (31.99%)	To explore the associations of short sleep with breakfast and snacking behaviors.	53.2% of children and adolescents consumed snacks (including SSBs), which was lower among participants with slightly short sleep (OR = 0.78; 95% CI: 0.68, 0.89) and severely short sleep (OR = 0.63; 95% CI: 0.49, 0.82) compared to those with moderate sleep after adjusting for the influence factors. The rate of consuming snacks at least once per day was 28.3%, and no significant difference among the three sleep groups was found. Among children and adolescents with severely short sleep, 8.7% choosed SSBs as snacks (204.7 g/day and 26.7 g/night).
	Ma L. et al. (2021) (37)	Cross-sectional and Longitudinal	China	3,298 6.5–17.5 y (11.5 ± 2.0) 6.5–12 y (1st-6th grade): 1,819 (55.2%) 12–17.5 y (7th-11th grade): 1,479 (44.8%) 1,691 6.5–16 y (11.2 ± 1.9)	To examine correlates of sleep and assess its associations with weight status and related behaviors.	Longer sleep was longitudinally associated with less SSBs intake (*β* = −0.12; 95% CI: −0.20, −0.03) after adjusting for covariates, such as age and sex. Sex-stratified analysis found longer SD to be associated with less SSBs consumption (*β* = −0.15; 95% CI: −0.28, −0.02) in boys.
	Min C. et al. (2018) (38)	Cross-sectional	Korea	118,462 12–18 y (15 ± 1.7)	To identify the associations between SD, SQ, and food intake.	Short SD (<6 h) were associated with a higher intake of soft drinks for ≥5 times a week (AOR = 1.73; 95% CI: 1.57, 1.91; *p* < 0.001). Soda intake showed an increasing trend in the group getting <6 h of sleep. Poor SQ was related with a higher intake of soda and soft drink for ≥5 times a week (AOR = 1.55; 95% CI: 1.40, 1.70 and AOR = 1.58; 95% CI: 1.43, 1.73 respectively; *p* < 0.001).
America	Cetiner O. et al. (2021) (50)	Cross-sectional	United States	1,556 12–17 y (14 (IQR: 13–16) in no SJL group, 14 (IQR: 13–16) in mild SJL group, 15 (IQR: 13–16) in severe SJL group)	To determine the association between SJL, and the frequency of consumption of multiple food and beverage groups and BMI percentile.	The median frequency of consumption of SSBs (per serving) was higher in the severe SJL (>2 h) group compared with the no SJL (<1 h) and mild SJL (1–2 h) groups (median 1.0 vs. 0.87 and 0.87; *p* < 0.001). Total sugar consumption and sugar consumption from SSBs (tsp) were higher in the severe SJL group (with a median value of 15.46 tsp. for total sugar, *p* = 0.004; and 5.34 tsp. for sugar from SSBs, *p* < 0.001) than in the no SJL and mild SJL groups (median total sugar consumption was 14.62 tsp. and 14.06 tsp. in the no SJL and mild SJL groups, respectively; and, median sugar consumption from SSBs was 4.2 tsp. in the no SJL and mild SJL groups).
	Egan K. A. et al. (2022) (39)	Pilot-site randomized trial	United States	100 9–12 y (10.1 ± 1.0)	To examine temporal associations between participation in a community-based intervention targeting sugary drink intake and sleep outcomes.	The intervention was associated with increased SD (*b* = 0.74; 95% CI: 0.03–1.45; *p* = 0.04) and higher odds of adequate sleep (OR = 2.47; 95% CI: 1.06–5.74; *p* = 0.04) at 2 months. Sleep did not differ by treatment condition at 6 months.
	Ievers-Landis C. E. et al. (2016) (51)	Cross-sectional	United States	315 13–18 y (14.5 ± 1.35)	To examine associations of SD and regularity with dietary intake and eating-related cognitions among adolescents with overweight and obesity.	Staying up (i.e., bedtime shift) (beta estimate = 0.23, *p* = 0.006) and sleeping in later (i.e., wake-time shift) (beta estimate = 0.59, *p* = 0.02) on weekends compared with weekdays significantly relates to drinking more SSBs, the latter for males.
	Lima R. J. C. P. et al. (2023) (53)	Cross-sectional	Brazil	2,515 18–19 y	To analyze the association between modifiable behavioral risk factors for NCDs and SQ and EDS, considering overweight as a mediator of these pathways.	The main modifiable behavioral risk factors for NCDs (physical inactivity, alcohol consumption, SSBs consumption, socioeconomic status) are associated with worse sleep indicators in adolescents, such as poor sleep quality and excessive daytime sleepiness, and are independent of overweight. Higher consumption of SSBs was associated with EDS (SC = 0.128; *p* < 0.001) and poor SQ (SC = 0.089; *p* = 0.003). Overweight was neither a mediator nor associated with SQ or EDS.
	Park S. et al. (2012) (40)	Cross-sectional	United States	16,188 14–18 y (9th–12th grade)	To examine the association of demographic characteristics, weight status, self-reported academic grades, and behavioral factors with sugar-sweetened soda intake.	The percentage of students who drank sugar-sweetened soda at least 1 time/day was higher among those who had <8 h of sleep on an average school night (*p* = 0.004). Sleeping <8 h on an average school night was significantly associated with sugar-sweetened soda intake at least 1 time/day during the past 7 days (OR = 1.18; 95% CI: 1.08–1.29).
	Sampasa-Kanyinga H. et al. (2018) (41)	Cross-sectional	Canada	9,473 11–20 y (15.2 ± 1.8) 11–13 y: 2,084 (22.0%) 14–17 y: 6,489 (68.5%) 18–20 y: 900 (9.5%)	To examine the relationship between SD and consumption of SSBs and EDs.	After adjusting for covariates, short SD was associated with greater odds of consuming SSBs in middle school students (OR = 1.64; 95% CI = 1.21, 2.24) but not those in high school (OR = 1.06; 95% CI = 0.86, 1.31). Short SD was associated with greater odds of EDs consumption in both middle (OR = 1.60; 95% CI = 1.10, 2.34) and high school (OR = 1.78; 95% CI = 1.38, 2.30) students after adjusting for sociodemographic factors. Although boys were more likely than girls to consume SSBs and EDs, the relationship between SD with consumption of SSBs and EDs did not differ by sex.
	Vézina-Im L. A. et al. (2024) (48)	Cross-sectional	Canada	218 14–17 y (15.3 ± 1.1)	To verify if the consumption of different beverages (e.g., water, 100% pure fruit juice, and SSBs) is associated with SQ.	The total water (*r*s = 0.09; *p* = 0.1630), 100% pure fruit juice (*r*s = 0.07; *p* = 0.2910), and SSBs (*r*s = −0.09; *p* = 0.1755) consumption levels were not significantly correlated with SQ in adolescents. Among caffeinated SSBs, EDs (*r*s = −0.16; *p* = 0.0197) and sugar-sweetened coffee (rs = −0.33; *p* < 0.0001) intake was correlated with adolescents’ SQ. EDs consumption (*β* = −0.0048; *p* = 0.0005) and being male (*β* = 0.6033; *p* < 0.0001) were associated with adolescents’ SQ. There was an interaction between sugar-sweetened coffee intake and biological sex that was associated with adolescents’ SQ (*p* = 0.0053). Sugar-sweetened coffee consumption was correlated with adolescent girls’ abilities to go to bed (*r*s = −0.21; *p* = 0.0203) and fall asleep (*r*s = −0.28; *p* = 0.0020), while in boys, it was only significantly correlated with their abilities to go to bed (*r*s = −0.27; *p* = 0.0069).
	Watts A. W. et al. (2018) (42)	Cross-sectional	United States	2,793 14.4 ± 2.0 y	To identify the most important factors within the personal, family/home, peer, school, neighborhood, and media contexts associated with SSBs intake, and to determine their contribution in explaining SSBs intake.	Results of individual regression analyses showed that more hours of sleep were associated with lower SSBs consumption (*β* = −0.07; SE = 0.02; *p* < 0.001). However, in the mutually adjusted model the association was no longer statistically significant.
	Weiss A. J. et al. (2023) (43)	Longitudinal with natural experiment design	United States	Baseline (Complete sample): 2,134 14–15 y (15.2 ± 0.3) (9th grade) Baseline (Policy sample): 1,267 14–15 y (15.1 ± 0.3) (9th grade) Baseline (Control schools): 867 14–15 y (15.2 ± 0.4) (9th grade)	To examine the effects of high school start time delay, a proven sleep-promoting intervention, on SSBs consumption	Mean baseline SSBs consumption was 0.9 (±1.5) beverages/day in policy change schools and 1.2 (±1.7) beverages/day in comparison schools. No evidence of impact of start time change on total SB consumption. Difference-in-difference estimates revealed a small decrease in the number of caffeinated SSBs consumed between baseline and the second follow-up period (after 2 y) in students attending the policy change schools relative to comparison schools in both crude (−0.11/day (−0.2165,−0.0011), *p* = 0.048) and adjusted analyses (−0.11/day (−0.2001,−0.0112), *p* = 0.028).
	Widome R. et al. (2019) (44)	Cross-sectional	United States	2,134 14–15 y (9th grade)	To assess how self-reported SD on school nights was associated with weight-related behaviors (eating, diet, and physical activity) and overweight/obesity.	Shorter SD was associated with more frequent consumption of sugar-sweetened, diet, caffeinated, and sports/energy beverages (p < 0.0001 for all items, in both unadjusted and adjusted models).
	Zhang K. et al. (2022) (52)	Cross-sectional	Canada	1,031 13–18 y (15.1 ± 0.7)	To assess whether greater SJL is associated with higher SSBs consumption in adolescents, and whether associations are stronger in one gender.	The odds of any SSBs intake were significantly higher in adolescents with SJL between 1 and 2 h (OR: 1.65; 95% CI:1.14, 2.38) and over 2 h (OR: 1.87; 95% CI: 1.11, 3.14), compared with 1 h or less; associations were stronger and only significant in girls. Girls with over 2 h of SJL had almost twice the odds of drinking any SSBs (OR:1.97; 95% CI: 1.06, 3.66), compared with girls with ≤ 1 h of SJL.
Europe	Kjeldsen J. S. et al. (2014) (45)	Cross-sectional	Denmark	676 8–11 y	To investigate association between objectively measured SD as well as parent-reported sleep problems and dietary risk factors for overweight and obesity.	SD was negatively associated with energy density (ED) of the diet (kJ/g) (β (95% CI) = −0.32 (0.54–0.11), p = 0.003), intake of sugar from SSBs (E%) [*β* (95% CI) = −1.50 (2.27–0.73), *p* < 0.001], and dietary energy from added sugar (E%) [*β* (95% CI) = −1.07 (1.61–0.54), *p* < 0.001]; SD variability was positively associated with intake of SSBs [β (95% CI) = 0.20 (0.03–0.38), *p* = 0.03], and the CSHQ score with ED [β (95% CI) = 0.16 (0.004–0.31], p = 0.04). ED, added sugar and SSBs were higher (*p* ≤ 0.03) among short sleepers (7.47–8.56 h/night; *n* = 169) compared with long sleepers (9.32–10.50 h/night; *n* = 168) when divided into quartiles.
	Kristjansson A. L et al. (2014) (55)	Cross-sectional	Iceland	11,132 10–12 y	To assess the prevalence of caffeinated SSBs consumption and examine the relationship between this consumption and common physical complaints.	For both genders, the prevalence of sleep problems increased with greater frequency of cola drink consumption. The AORs show a dose–response relationship between cola drink consumption and sleep problems only in boys (SSBs consumption ≥1/day vs. no consumption: OR: 1.31; 95% CI: 1.03, 1.67). For boys and girls the prevalence of sleep problems generally increased with greater EDs use. The AOR models show a dose–response relationship between EDs consumption and sleep problems only in boys (EDs consumption ≥1/day vs. no consumption: OR: 1.63; 95% CI: 1.25, 2.12).
Oceania	Matricciani L. et al. (2020) (49)	Cross-sectional	Australia	1,043 11–12 y (12.0 ± 0.4)	To determine sleep profiles of Australian adolescents and their parents and examine sociodemographic and lifestyle correlates.	Overall good sleeper’s consumed fewer sugary drinks, while participants with a late to bed sleep profile consumed higher amounts of sugary drinks (RR = 1.31; 95% CI: 1.02, 1.68; *p* = 0.034). Further analysis of constituent food group categories revealed statistically significant differences in cluster membership and adolescent’s consumption of EDs (RR = 0.28; 95% CI: 0.11, 0.71; *p* = 0.007).
	Morrissey et al. (2019) (56)	Cross-sectional	Australia	2,253 8.8–13.5 y (10.91 ± 1.1) ~9–10 y (4th grade):1,167 (51.8%) 11–12 y (6th grade):1,086 (48.2%)	To investigate the influence of lifestyle factors on children’s sleep dimensions and whether they modify the association between sleep and weight status.	Consuming two or more SSBs per day, or consuming an SSBs in the hour prior to bedtime, increased the odds of having 2 sleep problems (OR = 1.70; CI: 1.19, 2.43; and OR = 1.82; CI: 1.18, 2.80, respectively) or ≥3 problems (OR = 1.96; CI: 1.37, 2.80; and OR = 2.21; CI: 1.32, 3.72, respectively), compared with consuming an SSBs once per day and sometimes/never before bed.
	Nuss T. et al. (2021) (46)	Cross-sectional	Australia	8,942 12–17 y (14.7 ± 1.2)	To examine the patterns in consumption of EDs and identificate sociodemographic and behavioral correlates associated with regular consumption.	The factor most strongly associated with regular EDs consumption was consumption of other sugary drinks: students who reported drinking ≥4 cups/week of other varieties of sugary drinks being nearly four times as likely to also regularly consume EDs compared to students who usually drank ≤3 cups of other sugary drinks (OR = 3.81 (3.12–4.66) *p* < 0.001). Students who were not meeting sleep recommendations were more than twice as likely to be regular EDs consumers, compared to those who reported sleeping for at least the minimum recommended hours on a usual school night (OR = 2.10, 95% CI: 1.74–2.53, *p* < 0.001).
	Scully M. et al. (2017) (47)	Cross-sectional	Australia	7,835 12–17 y (8th-11th grade)	To examine demographic and behavioral correlates of high consumption of soft drinks and to explore the associations between high consumption and soft drink perceptions and accessibility.	Behavioral factors associated with high soft drink consumption (≥ 4 cups/week) were low fruit intake (≤1 serving/d) (OR = 1.70, 95% CI: 1.35–2.15, p < 0.001), consuming energy drinks ≥1 cup/week (OR = 2.01, 95% CI:1.59–2.54, *p* < 0.001), eating fast foods ≥1 times/week (OR = 2.36, 95% CI:1.98–2.80, *p* < 0.001), eating snack foods ≥14 times/week (OR = 2.58, 95% CI: 2.10–3.16, *p* < 0.001), watching television for >2 h/d (OR = 1.48, 95% CI:1.21–1.81, *p* < 0.001) and sleeping for <8 h/school night (OR = 1.34, 95% CI:1.09–1.65, *p* = 0.005)

EDs, Energy Drinks; BMI, Body Mass Index; SD, Sleep Duration; PA, Physical Activity; ST, Screen Time; AOR, Adjusted Odds Ratio; CI, Confidence Interval; SQ, Sleep Quality; SSBs, Sugar-Sweetened Beverages; SBs, Sugary Beverages; IQR, Interquartile Range; SJL, Social Jetlag; TSP, Teaspoon; CSHQ, Children’s Sleep Habit Questionnaire; NCDs, Non-Communicable Diseases; EDS, Excessive Daytime Sleepiness.

**Table 3:** Characteristics of the studies addressing mental health that were included in the review.

Continent	Articles	Study design	Country	Population Sample size (N) and age (mean ± standard deviation)	Objective	Main results
Asia	Bui C. et al. (2023) (59)	Cross-sectional	Taiwan	18,509 13 y (7th grade) and 16 y (10th grade)	To examine the association between the clustering of unhealthy behaviors and DSs.	Individuals exhibiting clustering of unhealthy behaviors were more likely (AOR = 1.53, 95% CI: 1.48–1.58, *p* < 0.001) to exhibit DSs than those who have no or only one unhealthy behavior. Frequent SSBs consumption significantly associated with DSs (OR = 1.35, 95% CI: 1.24–1.47, *p* < 0.001).
	Candrarukmi D. et al. (2025) (60)	Cross-sectional	Indonesia	1,413 10–17 y (14.48 ± 1.61)	To ascertain the prevalence of DSs and identify related risk factors.	Adolescents who consume SSBs more than three times/week (OR = 2.472; 95% CI: 1.623–3.765) were more likely to experience DSs.
	Chen D. et al. (2024) (83)	Cross-sectional	China	7,928 6–16 y 6–11 y: 4,962 (62.59%) 12 y: 2,966 (37.41%)	To find out the risk factors associated with BPs.	Student’s intake of SSBs is associated with BPs in Chinese children and adolescents (X^2^ = 46.11; p < 0.001). Students with a SSBs intake of ≥1 times/week (OR = 1.26; 95% CI: 1.10–1.45) were at significantly increased risks for BPs.
	Chen L. et al. (2025) (61)	Cross-sectional	China	47,520 12–18 y (15.12 ± 1.88)	To examine the associations of BRI and SSBs consumption with PSs.	The results of LRA showed that adolescents in the group consuming SSBs ≥4 times/week (OR = 1.32; 95% CI: 1.22–1.42) had a significantly higher risk of developing PSs (p < 0.001). Moderate intake (2–3/week) was associated with lower risk (OR = 0.91; 95% CI: 0.86–0.96). The results of ordered LRA showed that adolescents in the group with BRI quartiles of Q4 and SSBs consumption ≥4 times/week (OR = 2.01; 95% CI: 1.77–2.30) had the highest risk of developing PSs (p < 0.001). The same trend was observed in boys (OR = 1.77; 95% CI: 1.48–2.13) and girls (OR = 2.61; 95% CI: 2.13–3.20) (p < 0.001).
	Guo X. et al. (2024) (62)	Cross-sectional	China	5,307 6–16 y 6–12 y (1st-6th grade):3,768 (71.00%) 12–15 y (7th–9th grade): 1,539 (29.00%)	To identify direct causal factors and pathways leading to NSSI in children and adolescents using computational causal discovery and machine learning methods.	The prevalence of NSSI was higher among students who consumed SSBs (33.98% in the SSBs+ group vs. 29.04% in the SSBs- group). Analysis identified 9 nodes with direct causal relationships to NSSI, including SSBs. SSBs consumption is linked to elevated depression risks and increased NSSI likelihood.
	Jin Z. et al. (2024) (63)	Cross-sectional	China	22,868 10–19 y (14.6 ± 1.8)	To examine the individual and interactive associations between ST, SSBs, and DSs.	After adjusting for cofounding factors, ST on weekdays (OR = 2.22, 95% CI 1.97–2.51; OR = 1.69, 95% CI 1.51–1.90), ST at weekends (OR = 1.73, 95% CI 1.57–1.91; OR = 1.43, 95% CI 1.30–1.56) and SSBs (OR = 1.48, 95% CI 1.31–1.68; OR = 1.40, 95% CI 1.24–1.57) were found to be positively correlated with DSs. No age group difference could be found in the association of SSBs with DSs. Interaction models indicated that ST and SSBs in combination were related to greater odds of DSs. Compared with late adolescents, early adolescents had a higher probability of DSs when exposed to the joint effects.
	Khan A. et al. (2017) (64)	Cross-sectional	Bangladesh	755 13–16 y (14.26 ± 1.15)	To assess the prevalence and socio-demographic correlates of DSs.	High SDs intake (≥5/week) increased the odds of DSs (OR = 2.38; 95% CI: 1.06, 5.35; *p* = 0.035).
	Kim Y. et al. (2023) (90)	Cross-sectional	Korea	55,748 12–18 y	To examine the association between BSM and UEB.	Adolescents who underestimated their body shape were likelier to have UEB (AOR = 1.18; 95% CI: 1.11, 1.25; *p* < 0.001). Adolescents with high-level stress (AOR = 1.59; 95% CI: 1.43, 1.77) and depression (AOR = 1.29; 95% CI: 1.23, 1.36) showed more likely to have UEB and statistically significant (*p* < 0.001). The underestimation group of boys showed a higher likelihood of UEB (AOR = 1.16; 95% CI: 1.08, 1.26) than the accurate estimation group. Similarly, girls who underestimated their body shape were likelier to have UEB (AOR = 1.19; 95% CI: 1.09, 1.30) than the accurate estimation group (*p* < 0.001).
	Lin C-J, et al. (2024) (65)	Longitudinal	Taiwan	1,560 6–11 y (1st–6th grade, for depressive symptoms) and 12–17 y (7th–12th grade, for SSBs habits)	To examine the associations between childhood DSs trajectories and adolescent SSB-habit trajectories, and mediating role of sleep problems in these associations.	Children who had moderate-stable (AOR = 1.35; 95% CI: 1.04–1.77), high-stable (AOR = 2.01; 95% CI: 1.28–3.15), or increasing (AOR = 1.97; 95% CI: 1.26–3.06) trajectories of DSs relative to those in the low-stable group were significantly more likely to belong to the high-stable trajectory of SSBs than to the low-stable SSBs group. The Z-mediation test showed that sleep problems significantly mediated the associations between trajectories of childhood DSs and trajectories of SSBs during adolescence (all *p* < 0.05).
	Noor Z et al. (2025) (87)	Cross-sectional	Pakistan	377 14–19 y (16)	To evaluate the impact of academic stress on eating patterns, dietary preferences, and sleep duration.	A weak negative correlation was found between academic stress and both eating patterns (rho = −0.124, *p* < 0.05) and sleep duration (rho = −0.116, *p* < 0.05), indicating that higher academic stress was linked to unhealthy eating habits and reduced sleep duration in adolescents. Among female students, those with high levels of academic stress were 2.13 times more likely to consume beverages, including SDs compared to females with low stress levels (OR = 2.13, 95% CI: 1.03–4.41, *p* < 0.05). Females with moderate stress levels were 3.23 times more likely to consume beverages like colas and sodas compared to low-stressed counterparts (OR = 3.23, 95% CI: 1.24–8.40, *p* < 0.05).
	Qiu Q. et al. (2024) (66)	Cross-sectional	China	3,026 19–22 y	To investigate the current status of SSBs consumption, SQ, and ASs, and their associations.	The results of LRA showed that college students with SSBs consumption of 2–5 times/week (OR: 1.38; 95% CI: 1.12, 1.70) and SSBs consumption of ≥ 6 times/week (OR: 2.47; 95% CI: 1.98, 3.08) had a higher risk of ASs (*p* < 0.01). The results of ordered LRA showed that college students with SSBs ≥ 6 times/week and poor SQ (OR: 5.06; 95% CI: 3.75–6.83) had the highest risk of ASs (*p* < 0.001). College students in the SSBs ≥6 times/week and poor SQ group had a higher risk of developing ASs among boys (OR: 7.61; 95% CI: 4.94–11.71) than girls (OR: 3.26; 95% CI 2.13 ~ 5.00) (*p* < 0.001).
	Ra J. S. (2022) (67)	Cross-sectional	South Korea	24,006 15–18 y (10th-12th grade)	To identify the effects of combining the consumption of SSBs and fast foods on mental health, including stress, DSs, and suicidal ideation.	Compared to low SSBs consumption (reference), medium (AOR: 1.07; 95% CI: 1.00, 1.14) and high SSBs consumption were associated with increased stress (AOR: 1.20; 95% CI: 1.11, 1.29). In addition, high SSBs consumption was associated with increased DSs (AOR: 1.19; 95% CI: 1.09, 1.30) and suicidal ideation (AOR: 1.18; 95% CI: 1.05, 1.32) compared to the reference.
	Ra J. S. (2023) (68)	Cross-sectional	South Korea	21,046 15–18 y (10th–12th grade)	To identify the combinations of SSBs consumption, screen-based sedentary time, and sleep duration and their association with adolescents’ DSs and suicidal ideation.	After adjusting for biological, social, and psychological factors, high SSBs consumption was associated with greater DSs (AOR = 1.18; 95% CI: 1.08, 1.30) than low SSBs consumption. However, SSBs consumption was not associated with suicidal ideation.
	Tang N. et al. (2025) (69)	Cross-sectional	China	50,222 12–18 y (15.09 ± 1.89)	To analyze the association between SSBs consumption, MVPA duration, and PSs.	The results of LRA showed that adolescents with SSBs consumption >4 times/week (OR = 1.36; 95% CI: 1.25, 1.49) had a higher risk of prevalence of PSs (p < 0.001). The results of ordered LRA showed that the adolescents in the group with SSBs consumption >4 times/week and MVPA duration <30 min/day group (OR = 2.28; 95% CI: 1.98, 2.64) had the highest risk of the prevalence of PSs (*p* < 0.001). The same trend was observed in boys and girls.
	Wang Y, et al. (2022) (70)	Cross-sectional	China	6,120 19–22 y (20.16 ± 1.03)	To assess the association between SSBs consumption and PSs during the COVID-19 pandemic.	Compared to SSBs consumption of <2 times/week, college students with SSBs consumption of ≥2 times/week (OR = 2.96, 95% CI: 2.36–3.70) had a higher risk of PSs (*p* < 0.001). The same trend was found for emotional symptoms (OR = 4.31, 95% CI: 3.49–5.31; *p* < 0.001), behavioral symptoms (OR = 4.55, 95% CI: 3.73–5.54; *p* < 0.001), and social adaptation (OR = 3.99, 95% CI: 3.26, 4.91; *p* < 0.001) difficulties dimensions.
	Xu H. et al. (2019) (71)	Cross-sectional	China	14,500 10–20 y (14.9 ± 1.8)	To illustrate current eating pattern and to explore their association with PSs.	The SSBs pattern was significantly associated with higher risk of all PSs (psychopathological symptoms: AOR = 1.38; 95% CI: 1.23, 1.55; emotional problems: AOR = 1.34; 95% CI: 1.20, 1.49; conduct problems: AOR: 1.25; 95% CI: 1.13, 1.38; social adaptation problems: AOR = 1.56; 95% CI: 1.36, 1.78). A significant dose–response relationship was observed (*p*- trend < 0.01).
	Xu et al. (2020) (73)	Cross-sectional	China	14,500 10–20 y (14.9 ± 1.8)	To evaluate the association between co-consumption of FF and SSBs and self-reported mental health.	The β value increases as the quantile of SSBs, and co-consumption of SSBs with FF increases in the quantile regression model (p trend < 0.01). The β values of the higher quantile are significantly higher than that of the lower quantile. In the interaction model, FF and SSBs in combination were associated with greater odds of PSs (AOR = 1.90; 95% CI: 1.69, 2.12). The synergy index, relative excess risk of interaction, and attributable proportions were 1.86 (95% CI: 1.17, 2.96), 0.41 (95% CI: 0.20, 0.63), and 0.22 (95% CI: 0.11, 0.33), respectively.
	Xu H. et al. (2020) (72)	Cross-sectional	China	14,500 10–20 y (14.9 ± 1.8)	To examine the association between ST, FF, SSBs and DSs and evaluate the mediating effects of FFs and SSBs in the association between ST and DSs.	ST, FFs and SSBs, were more likely to be associated with DSs, and ORs (95%CI) was 1.075 (1.036–1.116), 1.062 (1.046–1.078) and 1.140 (1.115–1.166) in the model adjusted for sociodemographic variables (*p* < 0.001). The mediating effects of FFs and SSBs in the association between ST and DSs in adolescents are also evaluated. The direct effect of ST on DSs was 0.125; the total mediating effect is 0.034; the total effect was 0.159; the ratio of mediating effect to total effect was 0.214.
	Xu H. et al. (2023) (74)	Cross-sectional	China	31,856 17–25 y 17–18 y: 12,783 (40.13%) 19–20 y: 16,311 (51.20%) 21–25 y: 2,762 (8.67%)	To analyze the association between consumption of various SSBs and DSs.	After adjusting for sociodemographic variables and potential influencing factors of adolescent DSs in Generalized Linear Models, consumption of carbonated beverages (*β* = 0.10; 95% CI: 0.07–0.13; *p* = 0.000), tea beverages (*β* = 0.05; 95% CI: 0.01–0.08; *p* = 0.010), and milk tea (*β* = 0.08; 95% CI: 0.05–0.11; p = 0.000) was associated with DSs in college students. Stratified analysis by gender showed that carbonated beverages (*β* = 0.11; 95% CI: 0.07–0.15; *p* = 0.000) and milk tea (*β* = 0.07; 95% CI: 0.01–0.13; *p* = 0.021) consumption was associated with DSs in boys; and carbonated beverages (β = 0.09; 95% CI: 0.05–0.13; *p* = 0.000), tea beverages (*β* = 0.09; 95% CI: 0.04–0.13; *p* = 0.000), and milk tea (*β* = 0.08; 95% CI: 0.04–0.11; *p* = 0.000) consumption was associated with DSs in girls. The direct effect value of SSBs on DSs was 0.012 (*p* = 0.169), and mediating effect value of physical sub-health was 0.052 (*p* < 0.01). The total effect value was 0.064. The direct effect accounted for 18.7% of the total effect, and the mediating effect for 81.3% of the total effect.
	Yu C. J. et al. (2016)^89^	Case–control	Taiwan	332 (173 cases with ADHD and 159 non-ADHD controls) 4–15 y (8.9 ± 2 cases with ADHD and 9.2 ± 2.7 non-ADHD controls)	To analyze the association between SSBs consumption and ADHD.	Children with higher SSBs consumption had a greater risk of having ADHD. After adjusting for cofounding factors, the OR was 1.36 (95% CI: 0.61–3.05) for children who consumed 1–6 servings/week, and the OR was 3.69 (95% CI: 1.291–10.60) for children who consumed ≥7 servings/week.
	Zeng Y. et al. (2025) (75)	Cross-sectional and Longitudinal	China	7,829 10.6 ± 2.17 y	To investigate the associations between life changes during the COVID-19 lockdown, and DSs/ASs.	Cross-sectional analyses revealed that higher risks of DSs/ASs were associated with a decrease in SSBs consumption (OR = 1.57; 95% CI: 1.30, 1.90 and OR = 1.39; 95% CI: 1.14, 1.70, respectively). In longitudinal analyses, no new association was found between SSBs consumption and DSs/ASs, and the previously observed cross-sectional associations disappeared.
	Zhang Y. et al. (2021) (84)	Cross-sectional	China	30,188 6–18 y (12.44 ± 3.47) 6–12 y (1st–6th grade): 13,420 (44.50%) 12–15 y (7th-9th grade): 8,232 (27.30%) 15–18 y (10th-12th grade): 8,536 (28.30%)	To investigate the association between SSBs consumption, takeaway dietary pattern, and PBPs.	The top three SSBs in the intake frequency were milk beverages drinks (not milk), vegetable protein drinks, and fruit and vegetable juice drinks. More frequent intake of SSBs (OR = 2.23, 95% CI = 2.0–2.47, p < 0.01) and higher takeaway consumption (OR = 2.34, 95% CI = 1.81–3.03, p < 0.01) were associated with higher SDQ total difficulties scales. The same results about SSBs consumption were also found in emotional symptoms (OR = 1.73, 95% CI = 1.60–1.87, *p* < 0.01). When low and medium consumption of SSBs was compared, who have high SSBs intake were more associated with total difficulties score (OR = 3.10, 95% CI = 2.67–3.59, *p* < 0.01). The joint associations of SSBs and takeaway pattern with SDQ were stronger than the associations individually.
	Zhang Y. et al. (2022) (76)	Cross-sectional	China	11,787 20.51 ± 1.88 y ≤19 y: 3,860 (32.70%) 20–22 y: 6,426 (54.50%) 23 y: 1,501 (12.70%)	To examine the associations between factors related to COVID-19 measures and mental health symptoms.	Higher SSBs intake was correlated with higher DSs (Soda beverages: χ2 = 172.88; Tea beverages: χ2 = 157.10) and ASs (Soda beverages: χ2 = 115.70; Tea beverages: χ2 = 94.71). The students who reported higher consumption of SSBs (> 4 bottles/day) had higher DSs (Soda beverages: OR = 2.25, 95% CI: 1.14–4.429; Tea beverages: OR = 2.57, 95% CI: 1.28–5.15) and ASs (Soda beverages: OR = 2.71, 95% CI: 1.35–5.48; Tea beverages: OR = 2.92, 95% CI: 1.41–6.07).
	Zhang Y, et al. (2025) (77)	Cross-sectional	China	11,018 13–18 y (15.81 ± 1.60)	To explore the associations between SSBs consumption, and relative grip strength with PSs.	The prevalence of PSs was significantly higher in adolescents in the SSBs consumption of >4 times/week group (OR = 1.95, 95% CI: 1.69–2.24), using the SSBs consumption of <1 time/week group as the reference group (*p* < 0.001). Adolescents in the SSBs consumption of >4 times/week group and relative grip strength quartiles as Q1 group (OR = 2.77, 95% CI: 2.09–3.67) had the highest risk of PSs (p < 0.001).
	Zhao et al. (2025) (85)	Cross-sectional	China	1,126 9.53 ± 0.66 y	To investigate dietary habits status and their associations with EBPs in pre-teen children, as well as explore the mediating effect of child self-concept in the associations between healthy dietary habits and EBPs.	Children who had unhealthy SSBs intake were at an elevated risk of EBPs (OR = 1.41; 95% CI: 1.03, 1.95).
America	Dabravolskaj J, et al. (2023) (79)	Cross-sectional and longitudinal	Canada	24,274 13–18 y (14.8 ± 1.2) and 13–18 y (15.8 ± 1.2)	To examine individual and overall adherence to lifestyle recommendations and to assess the relationship between adherence to these recommendations and the severity of DSs and ASs.	Adherence to SSBs recommendation at baseline was associated with lower DSs and ASs at follow-up (*β* = −0.20; 95% CI: −0.34, −0.06 and *β* = −0.19; 95% CI: −0.31, −0.07, respectively). Males adhering to the SSBs recommendation had lower severity of both DSs and ASs (*β* = −0.22; 95% CI: −0.42, −0.03 and *β* = −0.19; 95% CI: −0.37, −0.01, respectively), whereas in females the association was observed only for ASs (*β* = −0.21; 95% CI: −0.38, −0.04).
	Dabravolskaj J. et al. (2024) (78)	Cross-sectional and longitudinal	Canada	13,887 12–18 y (14.9 ± 1.2) and 12–18 y (15.8 ± 1.2)	To examine the association of diet with DSs, ASs, and psychological wellbeing.	SSBs consumption was associated with greater severity of DSs (*β* = 0.04; 95% CI: 0.01, 0.06) and ASs (*β* = 0.02; 95% CI: 0, 0.05), and poorer psychological wellbeing (*β* = −0.03; 95% CI: −0.05, −0.01) at follow-up; associations were stronger in male students.
	Dennison-Farris M. et al. (2017) (80)	Cross-sectional	United States	121 7–13 y (10.5 ± 1.6) 7–9 y: 35 (28.90%) 10–13 y: 86 (71.10%)	To determine the relationship between DSs and obesogenic behaviors and whether self-efficacy mediates that relationship, independent of obesity.	Higher DSs were associated with higher diet soda intake (Beta + SE = 0.044 ± 0.017; *p* = 0.012). No significant association with other SSBs intake, considered alone or pooled. Dietary and physical activity self-efficacy were not mediators in the relationship between DSs and obesogenic behaviors.
	Kaidbey J. H. et al. (2022) (86)	Virtual single-arm intervention	United States	150 8–14 y (11.4 ± 2.0)	To examine children’s physical and emotional responses during 3 days of SDs cessation.	At baseline 59.4% of participants consumed <2 servings/day of SDs. 77% of participants reported some caffeinated SDs consumption, and approximately a quarter (*n* = 35) of the full sample reported consumption of caffeinated SDs daily. During SDs cessation, children reported physical and emotional improvements, including being less tired, angry, and annoyed; having less trouble sleeping; and less frequently arguing with others, getting in trouble, and getting mad. Unfavorable responses, such as mood disturbances and having less energy, were reported by some participants.
Europe	Jonsson et al. (2024) (88)	Cross-sectional	Sweden	3,692 11–15 y	To investigate the association between dietary behaviors, overweight/obesity, and mental health and wellbeing.	Consumption of ≤1 time/week of SSBs was associated with higher life satisfaction (OR = 1.27; 95% CI: 1.04–1.55), and a lower likelihood of experiencing two or more psychosomatic health complaints in a week (OR = 0.68; 95% CI: 0.60, 0.79) and school-related pressure (OR = 0.83; 95% CI: 0.72, 0.97).
Oceania	Smout S. et al. (2023) (81)	Cross-sectional	Australia	6,640 11–14 y (12.7 ± 0.5) (7th grade)	To investigate associations between key modifiable lifestyle behaviors (sleep; physical activity; fruit, vegetable and SSBs consumption; ST; alcohol use and tobacco use) and mental health.	Lower consumption of SSBs was associated with lower anxiety, depression and psychological distress symptomology (anxiety: F6,55,806 = 11.3352, *p* < 0.001; depression: F6,5,786 = 21.0525, p < 0.001; psychological distress: F6,5,812 = 13.0627, *p* < 0.001). The lowest mean scores were observed in those who did not drink SSBs, who had an average ASs score 24% lower (ΔMscore: 6.3, 95% CI: 3.8–8.8), an average depression score 49% (ΔMscore: 3.9, 95%CI: 2.6–5.1), and an average psychological distress symptom score 31% lower (ΔMscore: 2.6, 95% CI: 1.2–4.1) than those who drank ≥14 cups/week. In a model including all behaviors and adjusting for sociodemographic factors, the relationship with depression remained significant for moderate-to-vigorous physical activity (F7,4,623 = 2.2358, *p* = 0.03), fruit consumption (F7,4,623 = 2.3038, *p* = 0.02), SSBs consumption (F6,4,623 = 2.7934, *p* = 0.01), sleep (F15,4,623 = 14.9503, *p* < 0.001), ST (F16,4,623 = 5.7318, *p* < 0.001) and tobacco use (F1,4,623 = 9.9597, *p* = 0.002).
International	Walsh S. D. et al. (2020) (82)	Multi-center cross-sectional	37 Countries	32,884 15 y (15.51 ± 0.36)	To explore a contemporary and empirically based model of clusters of risk for adolescents and to examine how they are associated with adolescent mental wellbeing.	Between the factors, low social support and problematic Social Media Use showed the largest effect on Life Satisfaction (ORs = 2.167 and 1.330, respectively) and psychosomatic complaints (ORs = 1.687 and 1.386, respectively). However, sugary foods and drinks also showed significant effects on Life Satisfaction and psychosomatic complaints (ORs = 0.863 and 1.117, respectively).

DSs, Depressive Symptoms; AOR, Adjusted Odds Ratio; CI, Confidence Interval; SSBs, Sugar-Sweetened Beverages; OR, Odds Ratio; SBs, Sugary Beverages; BPs, Behavioral Problems; BRI, Body Roundness Index; PSs, Psychological Symptoms; LRA, Logistic Regression Analysis; NSSI, Non-suicidal self-injury; ST, Screen Time; SDs, Sugary Drinks; BSM, Body Shape Misperception; UEB, Unhealthy Eating Behaviors; SQ, Sleep Quality; ASs, Anxiety Symptoms; MVPA, Moderate-to-vigorous physical activity; FF, Fast Foods; ADHD, Attention Deficit/Hyperactivity Disorder; PBPs, Psychological and Behavioral Problems, SDQ, Strength and Difficulties Questionnaire; EBPs, Emotional and Behavioral Problems.

The age of participants in the studies included ranged from approximately 6–27 years, owing to the inclusion of both children and young adults in some cases.

Most of the articles (*n* = 33) were conducted in Asia, particularly in China (*n* = 20). The Americas contributed 15 studies, predominantly from the United States (*n* = 9), while fewer were reported from Oceania (*n* = 5) and Europe (*n* = 3). Additionally, one study was international, involving participants from 37 different countries.

Regarding health outcomes, slightly fewer studies investigated the association between SSBs consumption and sleep outcomes (*n* = 25) compared with those focusing on mental health outcomes (*n* = 32).

### 3.1 Sleep and sugar-sweetened beverage consumption

Overall, this scoping review included 25 studies on the relationship between sleep outcomes and SSBs. According to the RU-SATED model (31), sleep is recognized as a multidimensional construct characterized by six key dimensions, which were investigated across the included studies as follows: 16 on Sleep Duration (SD) (32–47), 1 on Sleep Efficiency (SE) (48), 2 on Sleep Timing (ST) (35, 49), 1 on Sleep Satisfaction (SS) (38), 4 on Sleep Regularity (SR) (45, 50–52), and 1 on Sleep-related daytime functioning (53). In addition, 5 studies assessed Sleep Quality (SQ) (33, 53–56), which reflects an overall evaluation across multiple sleep dimensions.

#### 3.1.1 Sleep duration

Most of the studies examining the relationship between SSBs or EDs consumption and SD indicate that regular or higher consumption of these beverages is associated with shorter sleep, compared with the recommendations of the American Academy of Pediatrics (AAP), which advise 9–12 h of sleep for children and adolescents aged 6–12 years and 8–10 h for those older than 12 years (57). Particularly noteworthy for their large sample sizes are studies conducted by Min et al. (38) and Huang et al. (34), respectively in Korea and China. The Korean study reported that adolescents sleeping less than 6 h per night were more likely to consume soft drinks at least five times per week, with soda intake showing a clear upward trend in the short-sleep group (38). In addition, the Chinese study found that adolescents consuming SSBs 4–5 times per week or more than five times per week had markedly higher odds of insufficient sleep compared with those consuming them less frequently (34).

EDs and sugar sweetened soda were also linked to insufficient sleep (32, 40, 41, 44, 46), with students not meeting sleep recommendations showing increased odds of regular consumption compared to peers with adequate sleep (46).

Highlighting the importance of moderating SSBs consumption to support healthy sleep patterns, the only interventional study—a community-based program targeting SSBs intake and sleep outcomes—reported increased SD and higher odds of adequate sleep after 2 months (39). However, the intervention effect was not maintained at 6 months (39). On the other hand, a longitudinal study examining the effects of high school start time delays—a proven sleep-promoting intervention—found small but significant decrease in caffeinated SSBs consumption between baseline and the second follow-up period (after 2 years) in students attending the policy-change schools, with no changes observed for total SSBs intake relative to comparison schools (43).

In contrast with the majority of findings, Liu et al. (36) reported that overall snack consumption, including SSBs, was lower among slightly short sleepers and severely short sleepers compared with those with moderate sleep. Moreover, when specifically considering the consumption of SSBs at least once per day, no significant differences were observed across SD groups. Nonetheless, the authors also reported that children aged 6–17 years with severely short sleep were more likely to select SSBs as snacks (8.7%) and consumed them more frequently, averaging 204.7 g during the day and 26.7 g at night. The cross-sectional studies by Watts et al. (2018) (42) and Boozari et al. (2021) (24) reported no statistically significant associations. In Watts et al. (2018) (42), individual regression analyses initially indicated that longer SD was associated with lower SSBs consumption. However, this association was no longer significant in the mutually adjusted model. Similarly, Boozari et al. (2021) (24) found no significant correlation between SD and SSBs intake, although mean daily SSBs consumption differed across sleep-duration categories.

Interestingly, Li et al. (35) examined the key mediating role of SSBs consumption in the association among SD, late chronotype, and weight gain, performing statistical mediation analyses. Results indicated that SSBs could act as potential mediators in the relationship between SD and BMI. Likewise, Kjeldsen et al. (45) reported inverse associations between SD and dietary energy density, sugar intake from SSBs as a percentage of energy, and energy intake from added sugars, suggesting an overall obesity-promoting dietary pattern.

With regard to gender differences in the association between SSBs consumption and SD, findings across studies were inconsistent. Ma et al. (37) reported, in sex-stratified analyses, that longer SD was associated with lower SSBs intake in boys. In contrast, Sampasa-Kanyinga et al. (41) found that although boys were more likely than girls to consume SSBs and EDs, the associations between SD and beverage consumption did not differ by sex.

#### 3.1.2 Sleep efficiency

SE – which measures sleep continuity by considering sleep latency, number of awakenings, time spent awake after sleep onset, and unplanned early awakening (31) - was evaluated in only one study, conducted by Vézina-Im et al. (48), in a cross-sectional sample of Canadian adolescents. The authors examined whether the consumption of various beverages, including SSBs, was associated with SQ, assessed using the validated short version of the Adolescent Sleep–Wake Scale. This instrument captures multiple components of SE, including ease of going to bed, falling asleep, resuming sleep after nocturnal awakenings, and returning to wakefulness in the morning.

Overall, total SSBs intake was not significantly correlated with SQ. However, caffeinated SSBs, particularly EDs and sugar-sweetened coffee, showed significant negative associations with adolescents’ SQ.

Further sex-stratified analyses revealed an interaction between sugar-sweetened coffee consumption and biological sex in relation to SQ. Among girls, sugar-sweetened coffee intake was correlated with greater difficulty going to bed and falling asleep, whereas in boys, the association was limited to difficulties going to bed.

#### 3.1.3 Sleep timing

Sleep timing refers to the placement of sleep within the 24-h day, typically operationalized through habitual bedtime and wake time (e.g., chronotype) (31). Evidence on ST in relation to SSBs consumption was limited, with only two studies addressing this dimension (35, 49). Both reported that later sleep patterns were associated with higher intake of SSBs.

In the study by Matricciani et al. (49), adolescents classified as good sleepers consumed fewer SSBs, whereas those with a late-to-bed profile showed significantly higher intake. This delayed sleep pattern was also linked to greater consumption of EDs.

Consistent with these findings, Li et al. (35) observed that a later chronotype was directly associated with higher SSBs consumption. The study further contributed by providing insights into the potential mediating role of SSBs in the relationship between chronotype and BMI, using mediation analyses. Results showed that SSBs partially explained this association, along with physical activity and psychological condition.

#### 3.1.4 Sleep satisfaction

SS, reflecting subjective perceptions of restfulness or sleep-related difficulties (31), was assessed only in the cross-sectional study by Min et al. (38), which examined associations between SD, SQ, and food intake. Participants who reported poor or very poor recovery from fatigue after sleep, a measure of SQ used to operationalize SS, showed a higher likelihood of consuming soda and soft drinks ≥5 times per week.

#### 3.1.5 Sleep regulatory

Sleep regularity was investigated in four studies, which collectively suggested that adolescents who frequently consumed SSBs tended to exhibit more irregular sleep patterns (45, 50–52). SR concerns the stability of sleep patterns across days, both in terms of timing and duration (31).

Most studies focused on SJL, a form of circadian misalignment resulting from discrepancies between weekday and weekend sleep–wake schedules (58). Both Cetiner et al. (50) and Zhang et al. (52) reported a dose–response association, with higher SSBs consumption observed in adolescents with greater SJL. Specifically, Cetiner et al. (50) found that those with >2 h of SJL showed higher median intake (1.0 servings/day) compared with those with <1 h or 1–2 h. Similarly, Zhang et al. (52) reported increased odds of any SSBs intake in adolescents with 1–2 h or >2 h of SJL, although associations were significant only in girls.

Ievers-Landis et al. (51) examined SJL through weekend delays in bedtime and wake time, finding that later bedtime and wake time were associated with higher SSBs intake, with the latter association observed only in males.

Finally, evidence from a cross-sectional study in Danish schoolchildren (8–11 y) also supports this association, indicating that greater variability in nightly sleep duration was associated with higher intake of SSBs (45).

#### 3.1.6 Sleep-related daytime functioning

Sleep-related daytime functioning refers to the ability to remain alert, energetic, and attentive throughout the day, without excessive sleepiness or fatigue, underscoring that sleep health extends beyond the nocturnal period (31). This dimension was investigated only in the cross-sectional study by Lima et al. (2023) (53), which assessed excessive daytime sleepiness (EDS) as an indicator of impaired alertness. Specifically, using a structural equation model (SEM), this study aimed to analyze the associations between modifiable behavioral risk factors for NCDs and sleep outcomes in adolescents, with overweight included as a potential mediator (53). Results indicated that higher SSBs consumption was significantly associated with greater EDS, independent of overweight, which neither mediated the association nor was directly related to EDS (53).

#### 3.1.7 Sleep quality

Five studies assessed overall SQ by considering multiple dimensions of sleep health. Across these investigations, adolescents with poorer SQ consistently reported higher consumption of both SSBs and EDs (33, 53–56).

Two small cross-sectional studies from Iran (33) and Malaysia (54) reported that SQ, measured with the validated Pittsburgh Sleep Quality Index (PSQI), was associated with higher SSBs intake.

Similar results were also obtained in two larger cross-sectional studies conducted in Iceland (55) and Australia (56), respectively. Kristjansson et al. (55) found a higher prevalence of sleep problems among high consumers of caffeinated SSBs in both genders, although the specific type of sleep problems was not detailed. Furthermore, a dose–response relationship was observed in boys between daily cola consumption or EDs intake and a greater occurrence of sleep problems.

Consistently, Morrissey et al. (56) reported that adolescents consuming ≥2 SSBs per day had increased odds of experiencing two sleep problems or ≥3 problems, compared with those consuming SSBs once per day or less frequently.

Finally, particularly noteworthy is the cross-sectional study by Lima et al. (53), which investigated the potential mediating role of overweight in the association between behavioral risk factors, including SSBs consumption, and SQ in a sample of Brazilian adolescents using SEM. Results showed that higher consumption of SSBs was associated with poorer SQ. However, despite the well-established link between overweight and sleep disturbances, excess weight did not emerge as a mediator in this association.

### 3.2 Mental health and sugar-sweetened beverage consumption

This review included 32 articles aimed at studying the relationship between SSBs consumption and mental health. Most of the studies (*n* = 25) were conducted in Asian countries. Psychological symptoms (PSs) in various forms were studied in 24 articles (59–82), while the remaining studies examined other related outcomes [behavioral and emotional problems: *n* = 4 (83–86), academic stress: *n* = 2 (87, 88), attention deficit/hyperactivity disorder (ADHD): *n* = 1 (89), body misperception: *n* = 1 (90)].

#### 3.2.1 Psychological symptoms

Studies focusing on PSs have used various validated tools designed to assess these symptoms as a whole or to investigate specific aspects, such as anxiety, depression, and their consequences.

Three recent cross-sectional studies (61, 69, 77), carried out in three different samples of Chinese adolescents, found that higher SSBs consumption (>4 times/week) was positively associated with a greater prevalence of PSs.

Wang and colleagues (70) assessed the association between SSBs consumption and PSs during the COVID-19 pandemic, observing that, compared to SSBs consumption of <2 times/week, college students with SSBs consumption of ≥2 times/week had a significantly higher prevalence of PSs. The same trend was found for emotional symptoms, behavioral symptoms, and social adaptation difficulties dimensions (70). Similar results were observed by Zhang et al. (76) in a web-based survey during the pandemic, who reported that students with higher consumption of SSBs (>4 bottles/day) had higher depressive symptoms (DSs) and anxiety symptoms (ASs). In contrast, another study (75), aimed at investigating the associations between life changes during the COVID-19 lockdown and DSs/ASs using both cross-sectional and longitudinal designs, showed that decreased SSBs consumption was associated with higher prevalence of these symptoms. These associations attenuated or disappeared 1 year later (77).

Three studies included in this review were conducted in the same sample of Chinese adolescents (71–73). It has been observed that the SSBs pattern was significantly associated with higher probability of all PSs (71), and that the co-consumption of Fast Foods (FFs) and SSBs was associated with greater odds of PSs. The authors also observed that screen time (ST), FFs and SSBs consumption were more likely to be associated with DSs, and that FFs and SSBs consumption might play a role of mediating variable in the association between ST and DSs (72). Specifically, using a Bayesian multiple mediation model, the direct, mediating, and total effects linking SSBs, ST and FFs consumption to DSs were examined, showing that FFs represented the main mediator, while SSBs contributed a smaller mediating role (72). Moreover, the chain mediation path involving ST, FFs and SSBs further strengthened the association with DSs (72). In another study included (63), multiplicative and additive interaction models were performed to estimate the interaction effects of ST and SSBs on DSs, highlighting that ST and SSBs in combination were related to greater odds of DSs. Compared to older adolescents, younger adolescents had a higher likelihood of DSs when exposed to the combined effects (63).

Four studies included reported that frequent SSBs consumption significantly increased the odds of DSs (59, 60, 64, 74). Specifically, Bui and colleagues (59) also observed that adolescents exhibiting clustering of unhealthy behaviors, including frequent SSBs consumption, insufficient physical activity, and screen-based sedentary behaviors, were more likely to exhibit DSs than those who have no or only one unhealthy behavior. On the other hand, the article by Xu et al. (74), also showed that the mediating effect of physical sub-health – considered as a chronic condition of unexplained deterioration in physiological function, halfway between health and disease, manifested by a certain degree of physiological dysfunction, insufficient physical activity, decreased adaptability and immune function, and poor mental health – accounted for 81.3% of the total effect in the mediating model of SSBs associated with DSs.

A cross-sectional study aimed at determining the associations between DSs and obesogenic behaviors in school-aged American Indian children (10.5 ± 1.6 years) (80), reported a significant relationship between DSs and diet soda consumption, meal skipping, and certain ST variables. No significant association with other SSBs intake (considered alone or pooled), physical activity, fruit and vegetable intake, and BMI percentile was found (80). A longitudinal analysis by Lin and colleagues (65), instead, showed that children who had moderate-stable, high-stable, or increasing trajectories of DSs, relative to those in the low-stable group, were significantly more likely to belong to the high-stable trajectory of SSBs than to the low-stable SSBs group. Additionally, the study identifies sleep problems as a mediating factor in these observed associations during adolescence (65).

Regarding severe depression and its associated consequences, one study analyzed Non-Suicidal Self-Injury (NSSI) (62), while two studies conducted in South Korea examined suicidal ideation, among other outcomes (67, 68). In the first article (62), the prevalence of NSSI was higher among students who consumed SSBs (33.98% in the SSBs+ group vs. 29.04% in the SSBs– group). Analysis identified 9 nodes with direct causal relationships to NSSI, including SSBs consumption. The latter was linked to elevated depression and increased NSSI likelihood. In 2022, Ra (67) noticed that, compared to low SSBs consumption (reference), high SSBs consumption was associated with increased stress, DSs, and suicidal ideation. In addition, combining high consumption of SSBs and low to high consumption of FFs might have dose-dependent negative effects on stress, DSs, and suicidal ideation in Korean adolescents (67). In 2023 the same author reported that high SSBs consumption was associated with greater DSs, but it was not associated with suicidal ideation (68). The effect of the combination of SSBs consumption, screen-based sedentary time, and SD on DSs and suicidal ideation was also observed (68).

Only one study focused exclusively on ASs, reporting that college students who consumed SSBs 2–5 times/week or ≥ 6 times/week showed a higher probability of experiencing ASs (66). It has been also observed that students with SSBs ≥ 6 times/week and poor SQ had the greatest odds of ASs (*p* < 0.001) (66).

Smout et al. (81) investigated the associations between key modifiable lifestyle behaviors and mental health, reporting that lower consumption of SSBs was associated with lower anxiety, depression and psychological distress symptomology; with the lowest mean scores observed in those who did not drink SSBs. In a model including all behaviors and adjusting for sociodemographic factors, the relationship with depression remained significant for moderate-to-vigorous physical activity, fruit consumption, SSBs consumption, sleep, ST and tobacco use (81).

ASs and DSs were examined together in the studies by Dabravolskaj et al., who analyzed data from the large longitudinal COMPASS (Cannabis, Obesity, Mental health, Physical activity, Alcohol, Smoking, and Sedentary behavior) study in Canada (78, 79). The authors observed that adherence to SSBs recommendation at baseline was associated with lower DSs and ASs at follow-up (79); and that SSBs consumption was associated with greater severity of DSs, and poorer psychological wellbeing at follow-up (78).

Finally, this review includes an article presenting the findings of a contemporary, large, cross-national representative sample of adolescents aged 15 years from 37 countries (82). A seven-factor model of risk (substance use and early sex, low social support, insufficient nutrition, bullying, sugary foods and drinks, physical health risk, and problematic social media use) for mental wellbeing was suggested (82). Among these factors, low social support and problematic social media use showed the largest effect on Life Satisfaction and psychosomatic complaints. Sugary foods and drinks were also significantly associated with both outcomes, although with smaller effect sizes (82).

#### 3.2.2 Academic stress

Two studies examined the relationship between SSBs consumption and school-related stress (87, 88).

Noor and colleagues (87) conducted a cross-sectional survey aimed at evaluating the impact of academic stress on eating patterns, dietary preferences, and SD in Pakistani adolescents. A weak negative correlation between academic stress and both eating patterns and SD was found, indicating that higher academic stress was linked to unhealthy eating habits and reduced SD in adolescents. Among female students, those with high levels of academic stress were 2.13 times more likely to consume beverages, including sugary drinks compared to females with low stress levels. Females with moderate stress levels were 3.23 times more likely to consume beverages like colas and sodas compared to low-stressed counterparts. For male students, the analysis demonstrated that those experiencing high levels of academic stress were significantly more likely to consume FFs.

The cross-sectional study conducted by Jonsson et al. (88) in a sample of Swedish adolescents, investigated the association between dietary behaviors, overweight/obesity, and mental health and wellbeing. The analyses by individual food group revealed that the consumption of SSBs once per week or less was associated with higher life satisfaction, and a lower likelihood of experiencing two or more psychosomatic health complaints in a week and school-related pressure.

#### 3.2.3 Behavioral and emotional problems

Behavioral and emotional problems (BPs and EPs), considered as two main dimensions of mental disorders (91), were examined in four included studies (83–86).

Chen and colleagues (83) conducted a cross-sectional study to identify the risk factors associated with BPs and EPs, using total BPs, internalizing and externalizing problems, and eight specific syndromes (92, 93) to capture the different dimensions of these issues. Findings showed that intake of SSBs is associated with BPs in Chinese children and adolescents. In particular, students with a SSBs intake of ≥1 times/week showed significantly higher odds of BPs. Similar results were observed by Zhao et al. (85), in a sample of Chinese pre-teen children. The scale used to detect children’s EBPs (Emotional Behavioral Problems) at home or school was classified into three subscales (i) neurotic behaviors that represent different aspects of emotional difficulties; (ii) anti-social behaviors that estimate the conduct problems (iii) mixed behaviors containing the rest of problematic behaviors, such as hyperactive (94, 95). It has been observed that the unhealthy SSBs intake was positively associated with EBPs. The last study conducted in Asia on these aspects was carried out by Zhang and colleagues (84). The authors assessed psychological BPs using the Strengths and Difficulties Questionnaire (SDQ), including hyperactivity problems, emotional symptoms, conduct problems, peer problems, and prosocial problems (96). More frequent intake of SSBs and higher takeaway consumption were associated with higher SDQ total difficulties scales. The same results relating to SSBs consumption were also found in emotional symptoms, conduct problems, peer problems, and prosocial problems, with the exception of hyperactivity. Compared with low and medium SSBs consumption, children and adolescents with high SSBs intake showed higher total difficulties scores. Moreover, the combined associations of SSBs and takeaway consumption with SDQ scores were stronger than those observed for either factor individually.

Finally, Kaidbey et al. (86) examined physical and emotional responses during 3 days of sugary drinks cessation, in a sample of children (ages 8–14 years), who reported habitual consumption of ≥12 ounces of sugary drinks daily in the United States. During the cessation, children reported physical and emotional improvements, including being less tired, angry, and annoyed, having less trouble sleeping, and less frequently arguing with others, getting in trouble, and getting mad. However, unfavorable responses, such as mood disturbances and having less energy, were reported by some participants.

#### 3.2.4 Other mental health-related outcomes

Body shape misperception (BSM) and Attention Deficit/Hyperactivity Disorder (ADHD) are studied in relation to SSBs consumption in two included studies (89, 90).

The article by Kim and colleagues (90) examined the association between BSM and Unhealthy Eating Behavior (UEB) among Korean adolescents in a cross-sectional study. The authors defined UEB as the high consumption frequency (>3 times/week) of at least one of the following items: caffeinated EDs, FFs, carbonated beverages, and SSBs. Adolescents who underestimated their body shape were likelier to have UEB. Adolescents with high-level stress and depression were more likely to have UEB. The underestimation group of boys showed a higher likelihood of UEB than the accurate estimation group. Similarly, girls who underestimated their body shape were likelier to have UEB than the accurate estimation group.

Yu and colleagues (89) conducted a case–control study in a sample of Taiwanese children, hypothesizing that children with ADHD drink more SSBs. The results showed a dose–response relationship between ADHD and SSBs consumption, indicating that children with higher SSBs consumption had a greater risk of having ADHD. Specifically, the adjusted OR showed that children who consumed ≥ 7 servings/week of SSBs had nearly 4-fold greater odds of having an ADHD diagnosis than the reference group. A logistic regression analysis excluding females suggested that boys with ≥ 7 servings/week of SSBs intake had a greater risk of having an ADHD diagnosis than the reference group.

## 4 Discussion

**Figure 2:**
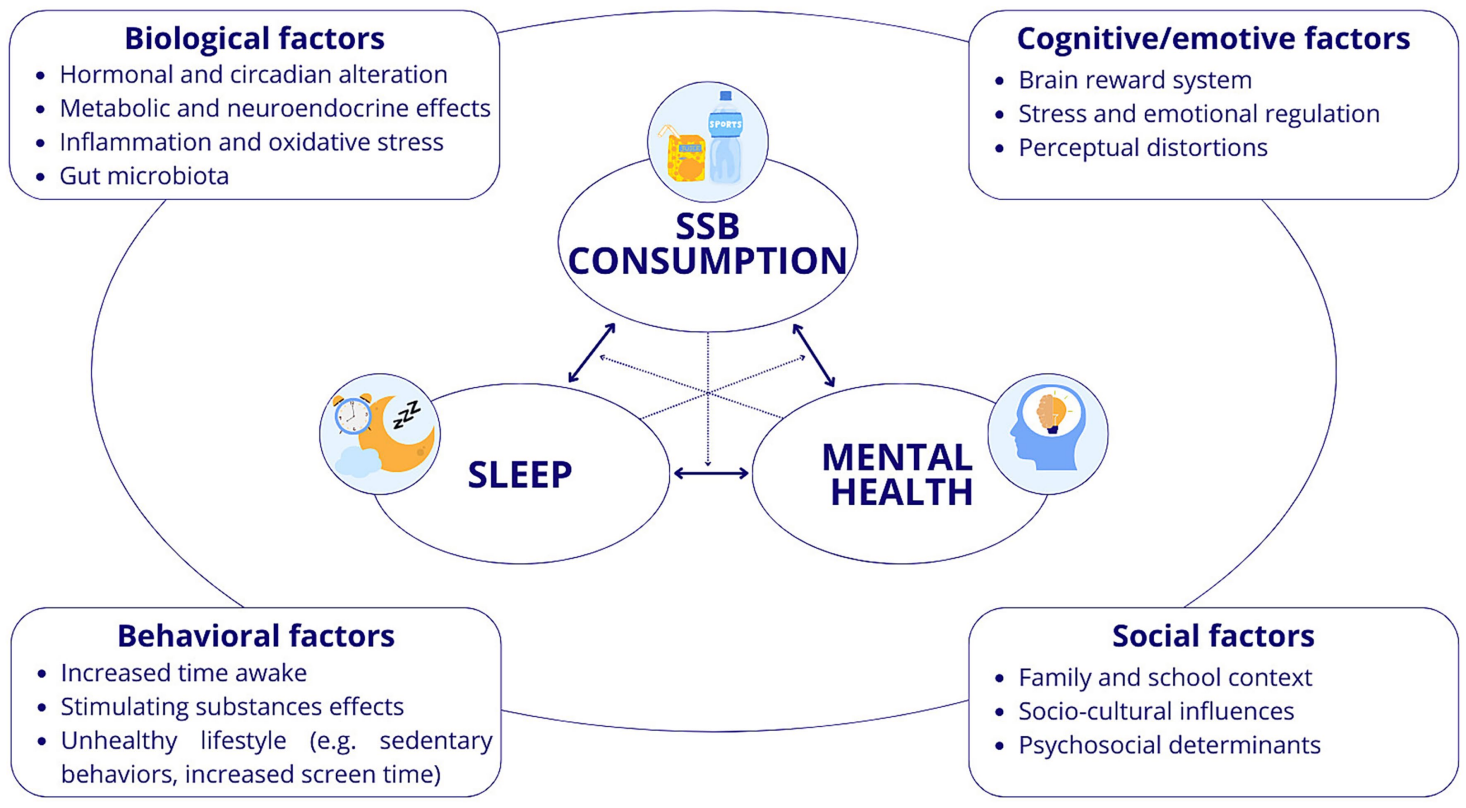
Potential mechanisms linking sugar-sweetened beverages, sleep, and mental health (created on https://www.canva.com/templates).

The findings of this scoping review suggest that higher SSBs intake is potentially associated with adverse sleep and mental health outcomes. These results are in line with recent systematic reviews reporting that frequent consumption of SSBs is linked to shorter SD, poorer overall SQ, and increased risk of depressive and anxiety symptoms (23–25, 97). However, these studies are largely descriptive, offering limited insights into the physiological pathways through which SSBs consumption may relate to sleep and mental health, which need to be further explored to better elucidate this relationship. A summary of the potential mechanisms proposed in the literature and adopted or advanced by the studies included to account for the observed associations is presented in Figure 2.

### 4.1 Potential mechanisms linking sleep and sugar-sweetened beverages

Several mechanisms have been proposed in the literature to explain the relationship between sleep patterns and food intake, including biological, cognitive, emotional, and behavioral aspects (98).

Disruptions of the circadian rhythm of appetite-related hormones and alterations in reward-related brain function were the mechanisms most frequently discussed across the reviewed studies, reflecting hypotheses primarily derived from the broader literature. Inadequate sleep seems to be associated with hormonal changes, such as decreased leptin and increased ghrelin levels, which may enhance appetite for energy-dense foods and beverages, including SSBs (34, 38, 49). Furthermore, short SD and poor SR during adolescence appear to be linked to circadian misalignment and dysregulation of reward-related neural responses to food stimuli, ultimately contributing to a greater preference for energy-dense and sugar-rich foods (45).

Another explanatory pathway proposed in the literature is that altered SD, SQ, and SR increase stress and fatigue, encouraging adolescents to intentionally consume SSBs or caffeinated SSBs to boost alertness, reduce sleepiness, and improve mood (34, 43). Moreover, insufficient sleep prolongs wakefulness, giving adolescents more opportunities to eat and drink, often leading to the consumption of easily accessible foods and beverages among youth in the late evening, when parental supervision is limited (37, 41, 54).

Possible explanations for the association between SSBs consumption and sleep outcomes were also hypothesized in the opposite direction, namely the potential impact of both SSBs and EDs on sleep. Several of the reviewed articles, consistent with broader literature, further elaborated on these proposed mechanisms. Caffeinated SSBs appear to play a central role in this mechanism, as their excessive intake, especially in the evening, may impair SQ. This, in turn, increases daytime sleepiness and encourages further consumption of these beverages, creating a self-reinforcing sleep–caffeine cycle (48, 50). Additionally, high sugar intake may exert stimulating effects, disrupting SD and SQ, increasing nocturnal awakenings, and contributing to excessive daytime sleepiness (34, 53).

Some of the studies included in this review have also attempted to clarify whether SSBs may mediate the relationship between sleep and obesity, showing inconsistent results due to the cross-sectional nature of available studies (35, 45, 53, 56). Although this remains an emerging and understudied line of research, findings from a systematic review suggest that adolescents with poorer sleep tend to consume more SSBs, which may partially explain their higher obesity risk (99).

Finally, within the studies reviewed, several have explored potential gender differences in the relationship between SSBs consumption and sleep outcomes, although results remain inconsistent across studies. Nevertheless, some evidence suggests that males may be more likely to increase SSBs intake in response to shifts in sleep timing, such as sleeping in on weekends. This may be related to the fact that males are more likely to be evening types, which could influence this particular association. However, the mechanisms underlying these gender differences remain unclear and require further investigation (51).

### 4.2 Potential mechanisms linking mental health and sugar-sweetened beverages

The association between eating behavior and mental health is complex and appears to be influenced by an interplay of psychological, biological, and social factors (71, 87). The cross-sectional design of most of the included studies does not allow causal inferences or deeper exploration of this association (83, 85, 87, 89). Importantly, adolescents’ mental health and wellbeing could shape their dietary behaviors; conversely, UEB may exacerbate poor mental health outcomes (72, 88).

Adolescents’ eating behaviors appear to be strongly shaped by psychological state. Acute stress can suppress appetite through cortisol release, leading to under-eating, whereas prolonged stress often promotes compensatory overeating and a preference for energy-dense foods (67, 87). More broadly, psychological wellbeing supports healthier food choices, while individual personality traits – including neuroticism and agreeableness – further modulate responsiveness to food and tendencies toward emotional eating (71). In this context, SSBs may function as a coping strategy to manage anxiety and stress (88). Coping style plays a pivotal moderating role: maladaptive strategies are linked to greater psychological distress, whereas cognitive and prosocial coping promote better mental health and wellbeing (76).

The evidence reviewed highlights several biological and neurobehavioral mechanisms - drawn from hypotheses proposed in the broader literature - through which SSBs consumption may influence PSs in adolescents. Excessive SSBs intake contributes to obesity, a known risk factor for poor mental health outcomes (61, 69, 77). SSBs alter gut microbiota composition, with downstream effects on hormonal regulation and brain function, potentially fostering emotional dysregulation (61, 69, 73). Moreover, high sugar intake promotes dopamine release, reinforcing consumption, and creating cycles of mood relief, appetite stimulation, and weight gain that increase psychological vulnerability (61, 70, 72). At the metabolic level, excessive fructose intake has been linked to insulin resistance, hypertension, and dyslipidemia, which not only increase cardiovascular risk but also activate the hypothalamic–pituitary–adrenal (HPA) axis, elevating glucocorticoids that are implicated in a spectrum of PSs (63, 70).

Across the included studies, there is a largely consistent hypothesis that unhealthy dietary patterns contribute to poor adolescent mental health through mechanisms involving oxidative stress, inflammation, and lack of micronutrients (e.g., B vitamins, omega-3 fatty acids, zinc, magnesium) essential for neural functioning and emotional regulation (64, 73, 74). Specifically, the combined consumption of FFs and SSBs appears to exert a synergistic effect, amplifying biological vulnerability to depression and anxiety (67, 73). The article by Zhang et al. (84) showed that both SSBs and takeaway dietary patterns independently and interactively increased the risk of psychological BPs: high SSBs consumption exacerbated the impact of frequent takeaway eating on PBPs. These UEB may be driven by environmental influences such as food advertising, ST, and home and school food availability, in line with the theory of planned behavior (100, 101). According to this framework, lifestyle changes influence not only dietary behaviors but also the cognitive processes underpinning them, with SSBs consumption interacting with other behaviors such as ST and physical activity in mutually reinforcing ways (76). In this context, UEB tends to cluster and reinforce one another, suggesting that interventions targeting a single behavior (e.g., reducing SSBs intake) could generate broader positive spillover effects (84).

Diet is one of the “Big 6” lifestyle domains shaping adolescent mental health, alongside physical activity, ST, sleep, tobacco, and alcohol use (81).

Evidence suggests that excessive SSBs consumption combined with low levels of moderate-to-vigorous physical activity (MVPA), heightens the prevalence of PSs via multiple pathways (69). Conversely, regular MVPA is potentially associated with better psychological wellbeing, enhanced academic outcomes, and greater self-confidence (69). Findings from rural China further indicate that high SSBs consumption combined with low muscle strength amplifies the prevalence of PSs, likely through reduced physical activity, inflammation, neuronal damage and obesity-related declines in strength (77).

It has been observed that SSBs consumption also interacts with ST, showing both multiplicative and additive effects on DSs in adolescents (63). Specifically, when ST is low, SSBs consumption is associated with an increased risk of DSs (multiplicative interaction), whereas when high ST and SSBs consumption coexist, the risk of DSs further increases (additive interaction) (63). While multiplicative interaction reflects statistical interaction and is more suitable for exploring potential causal mechanisms, additive interaction reflects biological interaction and is primarily used to inform public health priorities and intervention needs (63). Findings from Xu et al. (72) support the hypotheses that consumption of FFs and/or SSBs during ST may enhance the association with DSs in adolescents, with possible underlying explanations rooted in social, behavioral, and neuropsychological mechanisms, as suggested by other evidence in the literature. Moreover, differences reported in one of the included studies between weekday and weekend screen use highlight that weekend ST, often dominated by social media, is more strongly associated with DSs, likely due to reduced face-to-face interaction, social isolation, and exposure to cyberbullying (68, 80). ST may also serve as an avoidant coping strategy for existing DSs, reinforcing a negative cycle (80). Overall, these findings should be interpreted with caution due to the cross-sectional design of the studies.

The reviewed studies identify sleep as another potential mediator, aligning with hypotheses from the broader literature: DSs disrupt sleep regulation, and poor sleep in turn weakens prefrontal control over impulses, fostering reliance on high-sugar drinks as a form of emotional coping (65, 68). Excessive SSBs intake also disrupts circadian rhythms and hormonal balance, further impairing SQ and amplifying risks for anxiety and emotional instability (66). Both pathways converge on metabolic and neuroendocrine dysregulation—through cortisol, melatonin, and inflammatory responses—creating a cycle in which depression, poor sleep, and UEB reinforce one another (65, 66).

Overall, the results emphasize the importance of considering clustered risks in understanding adolescent mental wellbeing, and highlight the need for multidimensional, composite measures of risk to better identify vulnerable adolescents and guide prevention strategies (60, 63, 82).

Evidence from a short-term sugary drink cessation study (86) suggests that reducing habitual intake may lead to improvements in children’s BPs and EPs. Reported benefits included reduced irritability, oppositionality, restlessness, and sleep disturbances, with minimal withdrawal symptoms (86). Several mechanisms may explain these effects: more stable glycemia; better SQ; expectancy effects and social desirability; individual differences in habitual caffeine intake, sensitivity to sugar, and compensatory dietary behaviors (86).

Zhang and colleagues (84) did not observe a direct link with hyperactivity in their sample, probably due to beverage heterogeneity and offsetting effects. In contrast, one study included in this review indicated a dose–response relationship between ADHD and SSBs consumption (89). The potential impact of SSBs on ADHD and BPs/EPs has been attributed to multiple components in the literature, including sugar, artificial food colorings (AFCs), and preservatives. Proposed mechanisms for the adverse effects of sugar include: (a) sugar intolerance (physical discomfort after eating or drinking sugary foods); (b) body’s reactive hypoglycemia after ingestion; (c) decrease in intake of essential micronutrients; and (d) increased insulin released and brain serotonin concentration (linked to emotional dysregulation and later distress) (61, 84, 85, 89). However, reverse causality has also been suggested, whereby children with ADHD and BPs/EPs may have a greater propensity to consume SSBs (89). Evidence regarding AFCs and preservatives is inconclusive, and concerns remain, especially over azo dyes and compounds like sodium benzoate (89, 102). Given the multifactorial nature of BPs and the potential confounding role of food synergy, attributing these problems solely to SSBs intake is overly simplistic (83, 85, 89).

Finally, another interesting outcome was studied in relation to SSBs intake by Kim and colleagues (90). This study indicates that adolescents who underestimate their body shape are more likely to engage in UEB. Adolescents who misperceive their body shape are more likely to adopt unhealthy eating or weight control behaviors, driven by misconceptions or reduced motivation to adopt healthy habits or pursuit of an idealized body image (90, 103). Sociocultural factors, including media exposure and idealized body norms, contribute to body misperception, with girls tending to overestimate and boys to underestimate their body size, influencing sex-specific eating behaviors (64, 90). Importantly, in the study by Kim et al. (90) both sexes show a consistent association between underestimation of body shape and UEB, suggesting that interventions should target adolescents regardless of sex. However, given that most of the current evidence has sought to identify a relationship between exaggerated body size and mental problems or disordered eating focusing mainly on girls, future studies should determine sex-based associations between underestimating body shape and food consumption (90).

### 4.3 Strengths and limitations

To the best of our knowledge, this is the first review specifically focused on the relationship between SSBs intake, sleep, and mental health outcomes in adolescents, providing a comprehensive overview of the potential mechanisms currently available in the literature underlying these associations. Another strength is that, addressing adolescence, SSBs, and the various dimensions of sleep, the present work adopted detailed and widely shared definitions, considering established reference standards when available (29, 31).

However, the studies included present several limitations that warrant careful consideration. First, most of the studies had a cross-sectional design that does not allow for confirmation of causal relationships, highlighting the need for further randomized controlled trials and longitudinal cohort studies. Second, several studies included children and adolescents within the same sample, as well as individuals aged 18–19 years as part of an adult population, even though these age groups show biological, behavioral, and social differences that may distinctly influence the relationship under investigation. Moreover, these studies did not perform age-stratified analyses, thereby limiting the ability to draw adolescence-specific inferences. Third, most of the studies came from Asia, particularly China, followed by the Americas, whereas in countries that have recently seen an increase in SSBs consumption, the phenomenon remains largely unexplored. This may be related to a potential selection bias in this review, which only included articles in English. These patterns underscore the need to extend the search to evidence published in languages other than English and to conduct such investigations in currently underrepresented regions. Fourth, the lack of a standard definition of SSBs led to differences in how studies assessed these beverages, sometimes including or excluding certain types, making comparisons difficult. In addition, given the wide variety of beverages included in the SSBs group, it may be useful in future to conduct analyses by subgroup, in order to better clarify their potential effects. Fifth, most studies on sleep health focused on SD. Given that different dimensions of sleep interact with each other and contribute equally to overall sleep health (31), it would be desirable for future research to examine these other dimensions more thoroughly. Finally, most of the included studies assessed SSBs consumption and sleep and mental health outcomes through self-reported measures, contributing to potential recall, social desirability, and response biases.

## 5 Conclusion

Overall, the findings of this scoping review advance the hypothesis that higher SSBs intake may be involved in a potential bidirectional association with adverse sleep and mental health outcomes. The possible mechanisms linking SSBs consumption to sleep dimensions appear to be better described in the existing literature, whereas the pathways connecting SSBs intake with mental health outcomes remain less delineated due to their greater complexity and variability.

The main gaps identified in the currently available evidence warrant cautious interpretation of the findings and underscore the need for future studies that: adopt interventional and longitudinal designs; focus specifically on adolescence, in accordance with the WHO definition; are conducted in regions experiencing an increased prevalence of SSBs consumption, as well as sleep and mental health disorders; examine each SSB subgroup individually; and address all dimensions of sleep. Conducting research focused on specific age subgroups and establishing a shared definition of SSBs represent key priorities for improving study comparability.

The present review also emphasizes the importance of framing SSBs consumption not only as a nutritional concern but also as a behavioral and psychological risk factor in adolescence, highlighting the need for school- and community-based prevention strategies that adopt a comprehensive approach to fostering healthier environments.

## Data Availability

The original contributions presented in the study are included in the article/Supplementary material, further inquiries can be directed to the corresponding author.
