# Supplementary material for: How does sugar-sweetened beverage consumption relate to sleep and mental health in adolescents? A scoping review
Source: Front Nutr. 2026 Jan 12;12:1718230. doi: 10.3389/fnut.2025.1718230 (PMC12832302; doi:10.3389/fnut.2025.1718230)
Supplement: Supplementary file 1 [file Table_1.pdf]

# *Supplementary Material*

## 1 Table S1. Full systematic search strategy and search results implemented across four electronic databases.

| Database | Searches | Results |
|---|---|---|
| **Pubmed** | (("Sleep Hygiene"[Mesh]) OR ("Sleep Quality"[Mesh]) OR ("Sleep Duration"[Mesh]) OR ("Mental Health"[Mesh]) OR ("Sleep Disorders, Intrinsic"[Mesh]) OR "insomnia" OR "sleep disturbances" OR "psychological symptoms") AND (("Adolescent"[Mesh]) OR "adolescen*" OR "teenager" OR "youth") AND (("Sugar-Sweetened Beverages"[Mesh]) OR "sugary beverages" OR “sugary drinks”) | 10 |
| **Embase** | ('adolescent'/exp OR 'teenager' OR 'youth') AND ('sugar-sweetened beverage'/exp OR 'sugary beverage' OR 'sugary drink'/exp) AND ('sleep hygiene'/exp OR 'sleep quality'/exp OR 'sleep time'/exp OR 'mental health'/exp OR 'sleep disorder'/exp OR 'sleep disturbance' OR 'insomnia'/exp OR 'psychological symptom'/exp) AND [<1966-2025]/py | 74 |
| **Scopus** | ("adolescent" OR "teenager" OR "youth") AND ("sugar-sweetened beverage" OR "sugary beverage" OR “sugary drink”) AND ("sleep hygiene" OR "sleep quality" OR "sleep time" OR "mental health" OR "sleep disorder" OR "sleep disturbance" OR "insomnia" OR "psychological symptom") | 150 |
| **Web of Science** | (adolescent OR teenager OR youth) AND (sugar-sweetened beverage OR sugary beverage OR sugary drink) AND (sleep hygiene OR sleep quality OR sleep time OR mental health OR sleep disorder OR sleep disturbance OR insomnia OR psychological symptom) | 199 |

## 2 Table S2. Reasons for exclusion of articles from full-text screening.

| Articles | Reasons | | | | |
|---|---|---|---|---|---|
| | **Wrong population** | **Objective not relevant** | **Conference abstract only** | **Language** | **Full-text not available** |
| Bemanian M, et al. (2021) doi: 10.3390/ijerph18010130 | ✓ | | | | |
| Blouin J, et al. (2025) doi: 10.3390/nu17020217 | | ✓ | | | |
| Galy O, et al. (2020) doi: 10.3390/nu12072047 | | ✓ | | | |
| Grummon A, et al. (2021) doi: 10.1017/S1368980020002050 | ✓ | | | | |
| Gu W, et al. (2022) doi: 10.16835/j.cnki.1000-9817.2022.06.016 | | | | ✓ | |
| Hutchesson MJ, et al. (2021) doi: 10.3390/nu13020425 | | ✓ | | | |
| Ievers-Landis C, et al. (2015) doi: 10.1093/jpepsy/jsw017 | | | ✓ | | |
| Jiang JN, et al. (2023) doi: 10.3760/cma.j.cn112338-20230508-00286 | | | | ✓ | |
| Kelly N, et al. (2025) doi: 10.1007/s10865-024-00543-w | | | | | ✓ |
| Kim S, et al. (2017) doi: 10.5888/pcd14.160606 | | ✓ | | | |
| Komrij N, et al. (2021) doi: 10.1007/s12529-020-09876-7 | ✓ | | | | |
| Laska MN, et al. (2016) doi: 10.1016/j.ypmed.2016.06.001 | | ✓ | | | |
| Shih YH, et al. (2022) doi: 10.3389/fnut.2022.847704 | ✓ | | | | |
| Smout S, et al. (2021) doi: 10.1093/ije/dyab168.622 | | | ✓ | | |
| Smout S, et al. (2023) doi: 10.1016/j.anzjph.2022.100010 | | | ✓ | | |
| Song W, et al. (2024) doi: 10.3389/fpsyg.2024.1380893 | ✓ | | | | |
| Sun L, et al. (2025) doi: 10.3389/fpubh.2025.1554136 | | ✓ | | | |
| Tan S, et al. (2022) doi: 10.3389/fnut.2022.927212 | ✓ | | | | |
| Wang X, et al. (2025) doi: 10.16835/j.cnki.1000-9817.2025004 | | | | ✓ | |
| Ziegler AM, et al. (2015) doi: 10.5993/AJHB.39.6.3 | | ✓ | | | |
